# Supplementary material for: A causal role for the precuneus in network-wide theta and gamma oscillatory activity during complex memory retrieval
Source: eLife. 2019 Feb 11;8:e43114. doi: 10.7554/eLife.43114 (PMC6397002; doi:10.7554/eLife.43114)
Supplement: Figure 4—source data 1. — File contains data for panel C of Figure 4 (MTL-precuneus PAC), and data for non-significant mPFC and TPJ seeds. [file elife-43114-fig4-data1.zip › Fig4B-D_source/READ_ME.rtf]

T-values from surrogate-normalized cluster-corrected t-tests comparing phase-amplitude coupling between precuneus and vertex stimulation sessions for memory vs. rest. This folder contains 3 .csv files representing comparisons for the different neocortical regions of interest.Columns x Rows represent phase frequencies (3-7 Hz) x amplitude frequencies (65-85 Hz)
